# The Black Esophagus

**DOI:** 10.7759/cureus.18655

**Published:** 2021-10-11

**Authors:** Mathew Thomas, Vanessa Sostre Santiago, Fathima K Suhail, Guillermo Polanco Serra, Divey Manocha

**Affiliations:** 1 Internal Medicine, State University of New York Upstate Medical University, Syracuse, USA; 2 Gastroenterology, State University of New York Upstate Medical University, Syracuse, USA; 3 Gastroenterology, Geisinger Commonwealth School of Medicine, Scranton, USA

**Keywords:** proton pump inhibitors, endoscopy, anemia, necrotizing esophagitis, black esophagus

## Abstract

Acute esophageal necrosis (AEN), commonly referred to as black esophagus, is a rare clinical condition resulting from a combination of ischemic insult and thromboembolic injury to the esophagus. It is characterized by the circumferential black appearance of the esophagus. The risk factors for the development of AEN include coronary artery disease (CAD), diabetes mellitus, hypertension, malignancy, and alcohol use disorder. The treatment is directed at correcting the underlying medical conditions, supportive measures, and gastric acid suppression.

We present the case of a 60-year-old female with multiple medical comorbidities who was detected to have a black esophagus during the evaluation of anemia.

## Introduction

Black esophagus (necrotizing esophagitis) is an endoscopic description of acute esophageal necrosis (AEN), characterized by the diffuse circumferential black appearance of the esophageal mucosa. It is an extremely rare syndrome with a prevalence of 0.01%-0.2% [1]. The etiopathogenesis of AEN remains largely unclear, but ischemic insult and thromboembolic injury have been considered as important risk factors. Here, we report such a rare case of black esophagus detected incidentally for the evaluation of anemia in a 60-year-old female patient with significant cardiac history admitted to the medical intensive care unit (MICU) for septic shock secondary to urinary tract infection (UTI).

## Case presentation

Our patient is a 60-year-old female with a past medical history of coronary artery disease (CAD) status post percutaneous coronary intervention, ischemic cardiomyopathy (ejection fraction: 45%-50%), hypertension, hyperlipidemia, and squamous cell cancer of the cervix (patient refused surgery/chemotherapy) and a recent choledocholithiasis status post endoscopic retrograde cholangiopancreatography (ERCP) with common bile duct (CBD) stent placement. The patient presented to our hospital with generalized fatigue, weakness, postprandial abdominal pain, and decreased oral intake for two weeks. In the emergency department, she was afebrile, hypotensive with a blood pressure of 78/50 mmHg, tachycardic at 120 beats/minute, tachypneic at 22 breaths/minute, and saturating well on room air. Blood pressure did not respond to intravenous (IV) fluids; therefore, the patient was admitted to the MICU for vasopressor support. Further workup revealed urinary tract infection (UTI); hence, she was diagnosed with septic shock secondary to UTI and was started on empirical broad-spectrum antibiotics.

Her clinical condition improved, and she was subsequently transferred to the medical floors. Gastroenterology was consulted for the endoscopic evaluation of her worsening anemia (hemoglobin from 10.1 to 6.8 g/dL), intractable nausea, and vomiting without any signs of overt gastrointestinal (GI) bleeding.

**Figure 1:**
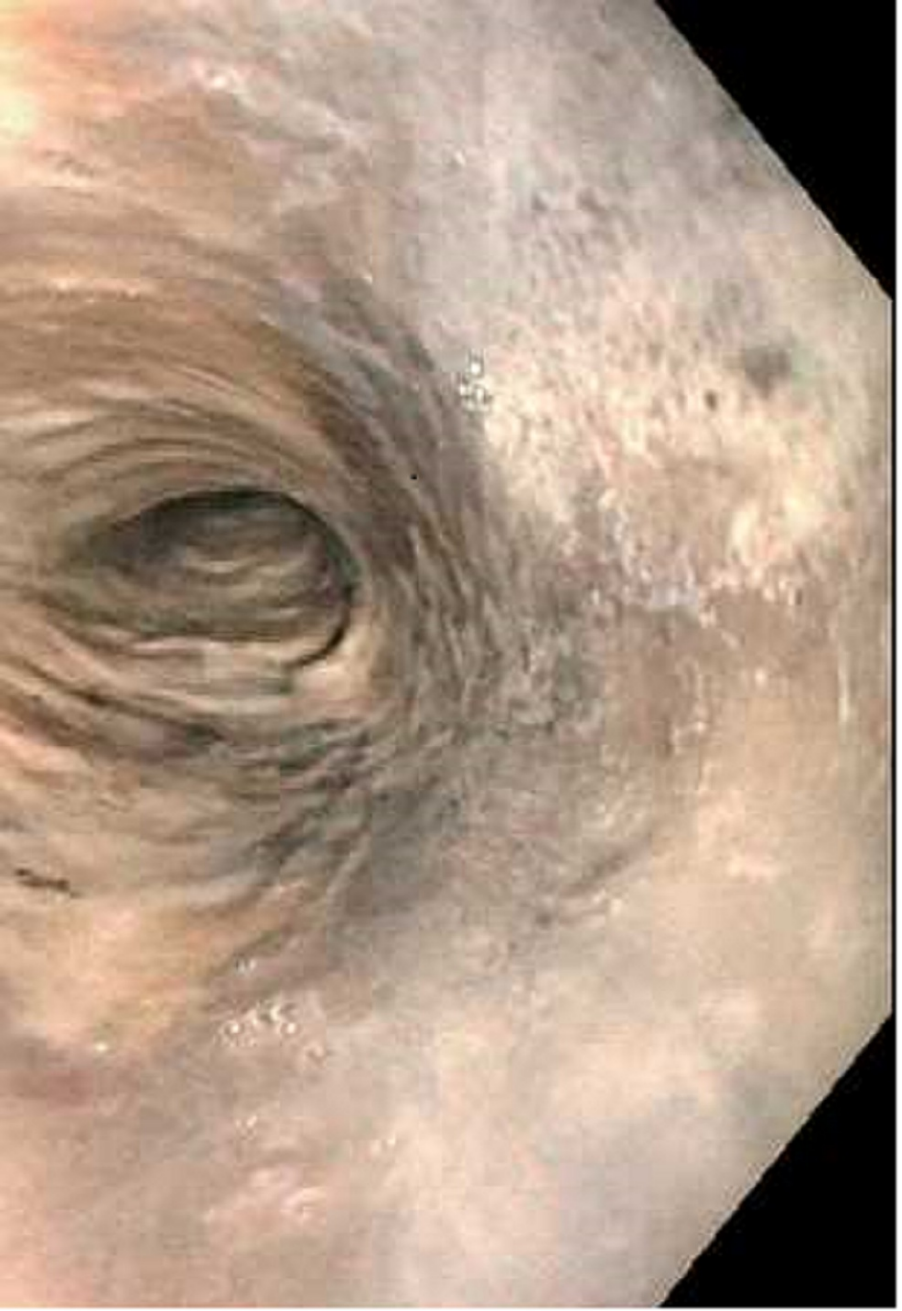
Black appearance of the esophagus in the middle third of the esophagus

**Figure 2:**
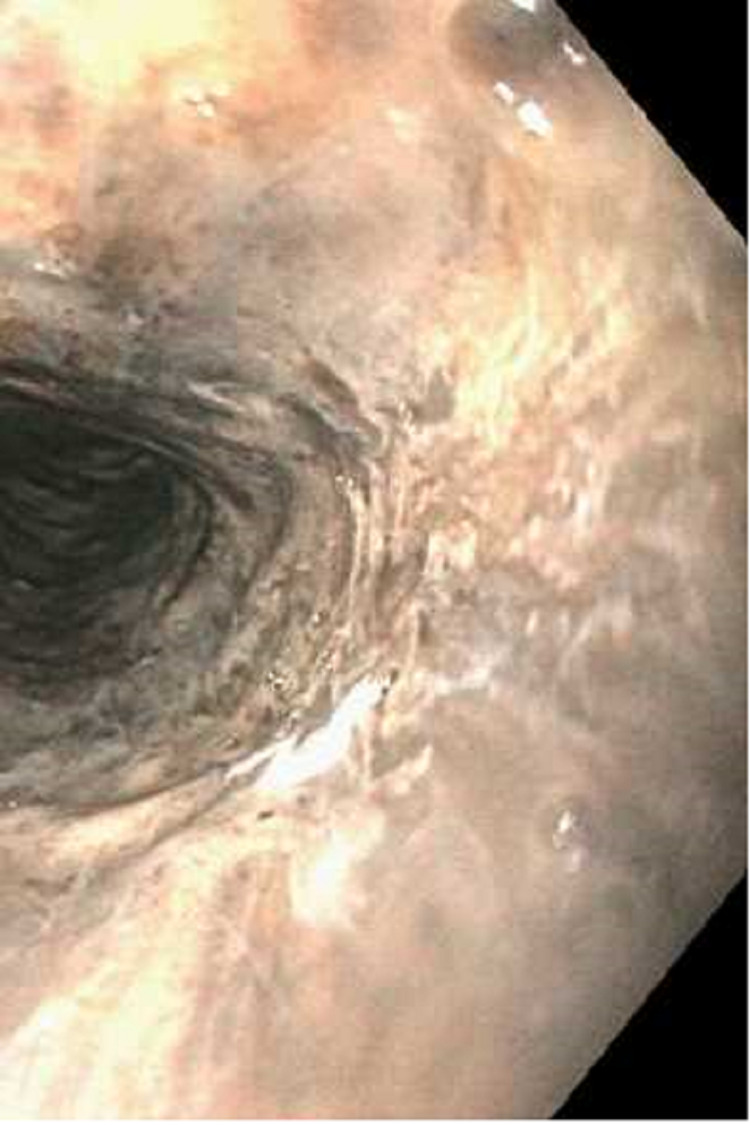
Black appearance of the esophagus in the lower third of the esophagus

**Figure 3:**
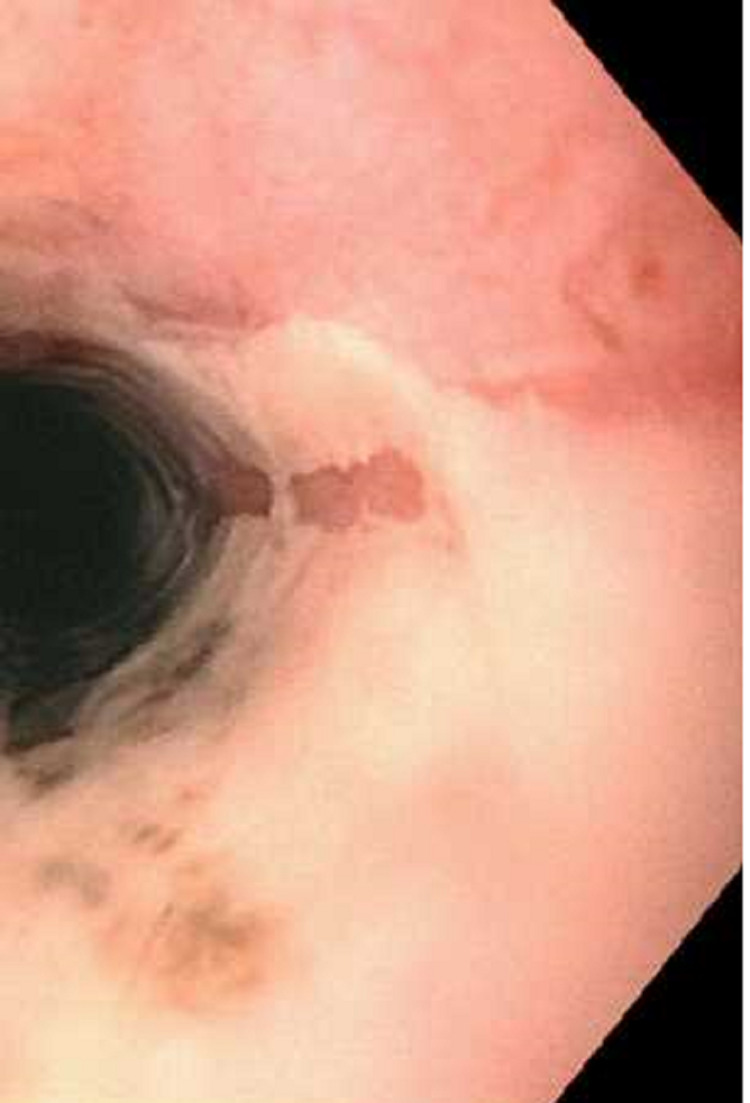
LA grade D esophagitis

**Figure 4:**
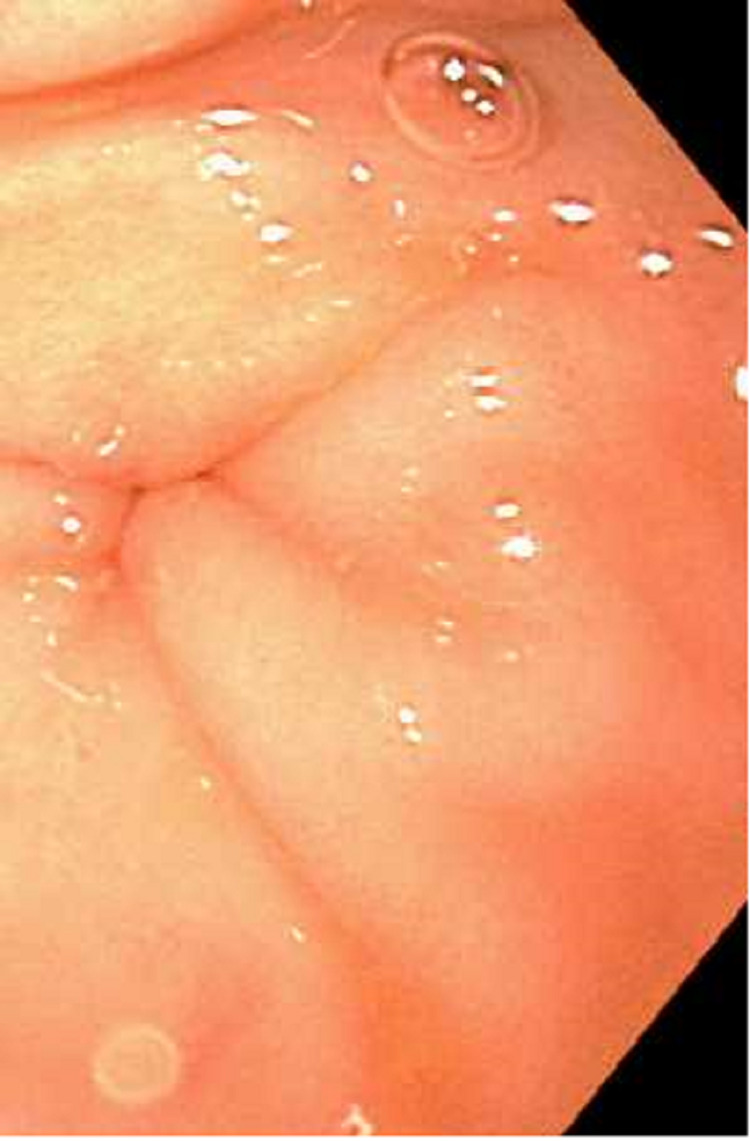
Normal gastric mucosa

**Figure 5:**
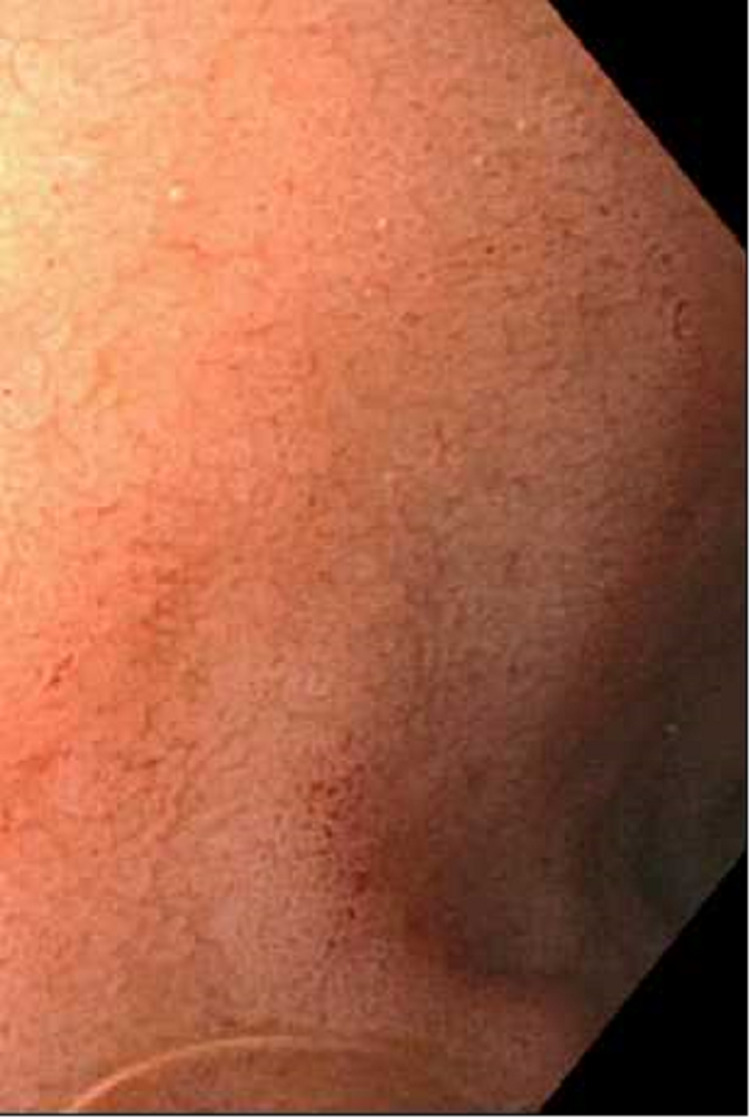
Mild erosions in the duodenal bulb

**Figure 6:**
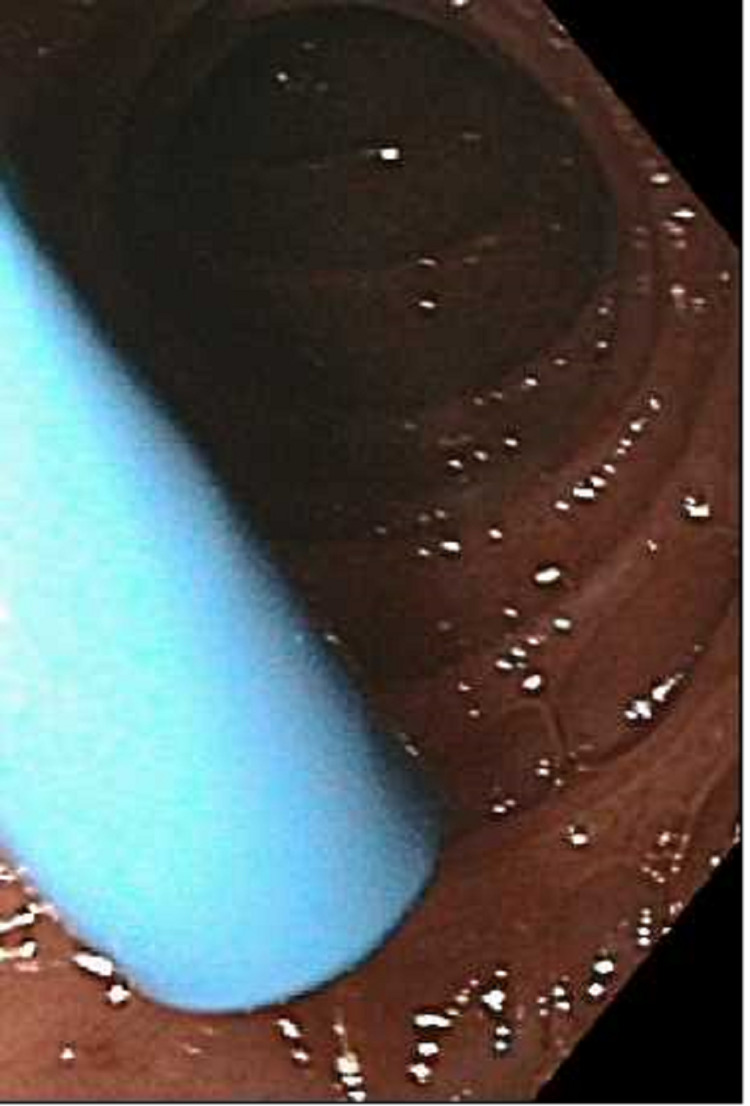
Normal-appearing second portion of the duodenum with the CBD stent placed during recent ERCP

Esophagogastroduodenoscopy (EGD) revealed severe mucosal changes in the middle and lower one-third of the esophagus, characterized by black discoloration (Figures 1 and 2), LA grade D esophagitis (Figure 3), normal stomach (Figure 4), mild erosions in the duodenal bulb (Figure 5), and a normal-appearing second portion of the duodenum with the patent CBD stent placed during recent ERCP (Figure 6). These esophageal findings were highly suspicious for acute esophageal necrosis and were not present during the recent ERCP performed three weeks ago.

Biopsies were not obtained due to the risk of perforation. She was started on IV proton pump inhibitor (PPI) twice daily and sucralfate 1 gram every six hours and placed on nil-per-oral (NPO) for one day. Her diet was advanced as tolerated. Her general condition improved over the following days, and she was discharged home on oral pantoprazole and sucralfate, with follow-up with her gastroenterologist for repeat EGD in three months to reassess for mucosal healing.

 

 

## Discussion

Acute esophageal necrosis (AEN) is a rare condition characterized by the diffuse circumferential black appearance of the esophageal mucosa [2]. AEN can affect any age group, but peak incidence occurs in the sixth decade of life [3]. AEN is considered to be multifactorial in origin. Tissue hypoperfusion and thromboembolic conditions play a significant role in the etiopathogenesis of AEN, including shock, sepsis, congestive heart failure, acute blood loss, hypothermia, coagulopathy, solid tumor or hematological malignancies, antiphospholipid antibody (APLA) syndrome, and atherosclerosis [4-6]. Patients with a history of coronary artery disease, diabetes mellitus, hypertension, malignancy, and alcohol use disorder are at increased risk of developing AEN [4-6].

Although it can affect a variable length of the esophagus, it most commonly affects the distal esophagus and spares the gastroesophageal junction as the lower esophagus has a lesser degree of vascularization compared with the proximal and middle portions [6,7]. Vascular compromise to the distal esophagus also explains the duodenal pathology commonly seen in AEN, namely, duodenal bulb ulcers, erosions, and inflammation [8]. Our patient had multiple risk factors that could have predisposed her to the development of AEN, including age; a history of diabetes, CAD, and cervical cancer; and most importantly the ischemic insult from the septic shock that required her to be on pressor support. She did not have any prior history of exposure to chemical agents. Our patient also had all the classical EGD findings of AEN as described above, including a black appearance in the distal one-third of the esophagus, gastric sparing, and erosions in the duodenal bulb. 

The important differential diagnosis of the black appearance of the esophagus includes malignant melanoma, melanosis, acanthosis nigricans, and coal dust deposition [2]. Esophageal perforation is the most serious complication of AEN, seen in <7% of cases, and should be suspected in rapidly deteriorating patients [3]. Perforation can lead to mediastinitis, mediastinal abscess, empyema, and sepsis. Other complications of AEN include bleeding, superinfection, and stricture/stenosis formation [7].

The treatment of AEN is focused on treating the underlying pathology, with adequate supportive care with volume resuscitation and blood and platelet transfusion (to maintain hemoglobin at >7 g/dL and platelet at >50,000/mm^3^). The patient should be initially kept NPO, and the diet should be advanced slowly. Patients should not be placed on a nasogastric tube to avoid perforation [9]. Medical management includes gastric acid suppression with proton pump inhibitors (PPIs) and mucosal protection with sucralfate [6]. Intravenous PPI is preferred initially and can be changed to oral form when adequate. Antimicrobial therapy is initiated for positive esophageal cultures and the presence of multinucleated giant cells or inclusion bodies on histopathological evaluation (if a biopsy is obtained) [7]. Surgical intervention in AEN is reserved for esophageal perforation with mediastinitis and abscess formation. Endoscopic balloon dilatation may be necessary for patients with symptomatic esophageal stenosis/stricture [6].

AEN carries a poor prognosis with a mortality rate of 30%-50% [7]. Endoscopic intervention with a differential of AEN should be considered in a patient with multiple risk factors, shock, and anemia even without overt GI bleeding, as seen with our patient. A repeat EGD should be done to reassess for mucosal healing.

## Conclusions

Black esophagus is characterized by the circumferential black appearance of the esophagus and results from a combination of ischemic insult and thromboembolic injury to the esophagus. Coronary artery disease, diabetes mellitus, hypertension, malignancy, and alcohol use disorder are considered to be the important risk factors for the development of black esophagus. Esophageal perforation is the most serious complication of black esophagus. The treatment includes gastric aid suppression and correcting the underlying medical condition. The mortality rate of black esophagus is high; hence, prompt initiation of treatment is of utmost importance.

## References

[1] Moretó M, Ojembarrena E, Zaballa M, Tánago JG, Ibánez S (1993). Idiopathic acute esophageal necrosis: not necessarily a terminal event. Endoscopy.

[2] Maher MM, Nassar MI (2008). Black esophagus: a case report. Cases J.

[3] Gurvits GE, Shapsis A, Lau N, Gualtieri N, Robilotti JG (2007). Acute esophageal necrosis: a rare syndrome. J Gastroenterol.

[4] Yasuda H, Yamada M, Endo Y, Inoue K, Yoshiba M (2006). Acute necrotizing esophagitis: role of nonsteroidal anti-inflammatory drugs. J Gastroenterol.

[5] Hawari R, Pasricha PJ (2007). Esophageal infarction. Curr Treat Options Gastroenterol.

[6] Gurvits GE (2010). Black esophagus: acute esophageal necrosis syndrome. World J Gastroenterol.

[7] Gurvits GE, Cherian K, Shami MN (2015). Black esophagus: new insights and multicenter international experience in 2014. Dig Dis Sci.

[8] Ben Soussan E, Savoye G, Hochain P, Hervé S, Antonietti M, Lemoine F, Ducrotté P (2002). Acute esophageal necrosis: a 1-year prospective study. Gastrointest Endosc.

[9] Ullah W, Abdullah HM, Rauf A, Saleem K (2018). Acute oesophageal necrosis: a rare but potentially fatal association of cocaine use. BMJ Case Rep.

